# Prenatal Protein Malnutrition Leads to Hemispheric Differences in the Extracellular Concentrations of Norepinephrine, Dopamine and Serotonin in the Medial Prefrontal Cortex of Adult Rats

**DOI:** 10.3389/fnins.2019.00136

**Published:** 2019-03-05

**Authors:** David J. Mokler, Jill A. McGaughy, Donna Bass, Peter J. Morgane, Douglas L. Rosene, Ana C. Amaral, R. Jarrett Rushmore, Janina R. Galler

**Affiliations:** ^1^Department of Biomedical Sciences, College of Osteopathic Medicine, University of New England, Biddeford, ME, United States; ^2^Department of Psychology, University of New Hampshire, Durham, NH, United States; ^3^Department of Anatomy and Neurobiology, Boston University School of Medicine, Boston, MA, United States; ^4^Department of Neurology, Massachusetts General Hospital, Harvard Medical School, Boston, MA, United States; ^5^Division of Pediatric Gastroenterology and Nutrition, Mucosal Immunology and Biology Research Center, MassGeneral Hospital for Children, Boston, MA, United States; ^6^Department of Psychiatry, Harvard Medical School, Boston, MA, United States

**Keywords:** 5-hydroxytryptamine, *in vivo* microdialysis, neurotransmission, 5-HT, infralimbic – prelimbic cortex

## Abstract

Exposure to prenatal protein malnutrition (PPM) leads to a reprogramming of the brain, altering executive functions involving the prefrontal cortex (PFC). In this study we used *in vivo* microdialysis to assess the effects of PPM on extracellular concentrations of norepinephrine (NE), dopamine (DA) and serotonin (5-HT) bilaterally in the ventral portion of the medial prefrontal cortex (vmPFC; ventral prelimbic and infralimbic cortices) of adult Long-Evans rats. Female Long-Evans rats were fed either a low protein (6%) or adequate protein diet (25%) prior to mating and throughout pregnancy. At birth, all litters were culled and fostered to dams fed a 25% (adequate) protein diet. At 120 days of age, 2 mm microdialysis probes were placed into left and right vmPFC. Basal extracellular concentrations of NE, DA, and 5-HT were determined over a 1-h period using HPLC. In rats exposed to PPM there was a decrease in extracellular concentrations of NE and DA in the right vmPFC and an increase in the extracellular concentration of 5-HT in the left vmPFC compared to controls (prenatally malnourished: *N* = 10, well-nourished: *N* = 20). Assessment of the cerebral laterality of extracellular neurotransmitters in the vmPFC showed that prenatally malnourished animals had a significant shift in laterality from the right to the left hemisphere for NE and DA but not for serotonin. In a related study, these animals showed cognitive inflexibility in an attentional task. In animals in the current study, NE levels in the right vmPFC of well-nourished animals correlated positively with performance in an attention task, while 5-HT in the left vmPFC of well-nourished rats correlated negatively with performance. These data, in addition to previously published studies, suggest a long-term reprogramming of the vmPFC in rats exposed to PPM which may contribute to attention deficits observed in adult animals exposed to PPM.

## Introduction

Protein malnutrition is one of the most prevalent forms of malnutrition in the world. Studies in human populations exposed to prenatal protein malnutrition (PPM) during the Dutch Famine have shown an increased lifetime risk of depression and schizophrenia (Susser et al., 1998; St Clair et al., 2005). The 45+ year Barbados Nutrition Study has documented cognitive and emotional development across the lifespan associated in a cohort that suffered from moderate-severe malnutrition limited to the first year of life and healthy comparison cases (Galler et al., 2012; Waber et al., 2014). Although they caught up completely in physical growth, the previously malnourished cohort displayed reductions in IQ, cognitive flexibility, impaired visuospatial processing, as well as problems with impulsivity and attentional dysregulation (Galler et al., 2012; Waber et al., 2014, 2018). Cognitive and attentional problems (Galler et al., 2012), including cognitive rigidity and poor cognitive control, led to poor performance on a national high school entrance exam (Galler et al., 1990); persisted at least to 40 years of age and were are also seen in the offspring of the original study participants (Waber et al., 2018). These attentional problems were closely associated with epigenetic changes in both the parent and offspring generation and may represent a mechanism underlying the long-term effects of early protein malnutrition (Peter et al., 2016).

Depression, schizophrenia, cognitive impairment, and attentional problems involve executive functions modulated by the prefrontal cortex (PFC) (Fuster, 2001; Robbins and Arnsten, 2009; Bagot et al., 2016). Furthermore, these disorders have been linked to imbalances in neurotransmitter systems in the PFC [See Robbins (2000) and Robbins and Arnsten (2009) for reviews]. In previous studies we have shown in a rat model of PPM that there are extensive changes in the serotonergic (5-HT) systems of the brain including an increase in 5-HT in the hippocampus of both 30-day old and adult rats (Mokler et al., 1999, 2003). Other studies using this model have shown that animals exposed to PPM have decreases in the dendritic arborization of serotonergic raphé cells and decreases in serotonergic terminal densities in the hippocampus as reflected by decreases in serotonin transporters (SERT) and 5-HT1A receptors (Blatt et al., 1994). We have also examined the effects of PPM on dopamine (DA) and shown, using *in vivo* microdialysis, that there is a decrease in the extracellular concentration of DA in the ventral medial prefrontal cortex (vmPFC) of adult rats following PPM (Mokler et al., 2007). Other laboratories have also reported on changes in 5-HT and DA in the brains of prenatally malnourished animals [see Almeida et al. (1996a) and Alamy and Bengelloun (2012) for reviews].

Although cognitive rigidity associated with malnutrition has been reported in humans (Waber et al., 2014) and in preclinical studies (McGaughy et al., 2014), which has been consistently linked to hypofunction of prefrontal norepinephrine (NE) (Tait et al., 2007; Newman et al., 2008; Mokler et al., 2017); the role of prefrontal NE in the cognitive deficits resulting from PPM are less clear. In the present study we used *in vivo* microdialysis to assess the effects of PPM on NE as well as 5HT and DA in the ventral portion of the medial prefrontal cortex. We focused on the ventral portion of the mPFC which consists of the ventral prelimbic and infralimbic PFC as defined in the atlas of Paxinos and Watson (2005). This distinction of the ventral mPFC is based on a functional view of the PFC discussed in more detail in Morgane and Mokler (2006). Based on our previous work showing differences in extracellular levels of these monoamines in the left and right cerebral hemispheres (Staiti et al., 2011), here we assessed laterality of concentration for all three monoamines using dual probe microdialysis. Our hypothesis is that exposure to PPM alters the pattern of 5-HT, NE, and DA levels in the left and right hemispheres of the medial PFC. In a subset of animals, we compare these monoaminergic changes to prior performance in an attentional task designed to assess cognitive flexibility as an initial step in linking changes in cortical neuromodulators to cognition.

## Materials and Methods

### Subjects and Housing Conditions

Long-Evan hooded rats were obtained from Charles River (Wilmington, MA, United States). They were housed in animal quarters maintained at a temperature of 23°C (±2°) and at 45–55% humidity with a reverse 12 h night (0700–1900) 12 h day (1900–0700) light cycle. Microdialysis experiments occurred during the dark phase of the cycle between 0900 and 1300 h, enabling observations during the active waking period of the rat and allowing for measurement of neurotransmitters during the same diurnal period as behavioral testing [as reported by McGaughy et al. (2014)]. Red fluorescent lighting in the animal room during the dark phase of the cycle provided continuous dim illumination for animal care and testing. All procedures described in this paper were approved by the University of New England Institutional Animal Care and Use Committee (protocol 20101005MOK) in accordance with guidelines outlined in the NIH Guide for the Care and Use of Laboratory Animals and the Society for Neuroscience Policies on the Use of Animals and Humans in Neuroscience Research.

### Nutritional Treatment and Breeding

This model of PPM implements dietary restriction prior to and during pregnancy with nutritional rehabilitation commencing at birth. Virgin female Long-Evan hooded rats were randomly assigned to one of two nutritional conditions. One group of females was fed an adequate protein diet (25% casein, Teklad Laboratories, Madison, WI, United States) beginning 5 weeks prior to mating and continuing throughout pregnancy, while the second group received an isocaloric, low protein diet (6% casein, Teklad Laboratories, Madison, WI, United States). These diets have been described in detail elsewhere (Tonkiss and Galler, 1990; Tonkiss et al., 1990) and in this issue (McGaughy et al., this issue). All females were mated with males that had been acclimated to the same diet for 1 week. Throughout pregnancy, dams were singly housed in individual polysulfone breeding cages, 39.5 cm × 34.6 cm × 21.3 cm (l × w × h; Tecniplast, Maywood, NJ, United States). Following parturition, litters from both nutritional groups were culled to eight pups (two females and six males) and were fostered as whole litters within 24 h of birth to foster dams receiving the 25% casein diet that had given birth within the same 24 h period. Pups born to mothers provided with the 6% casein diet were fostered to mothers on the 25% casein diet and designated as members of the 6/25 (prenatally malnourished) group, while pups born to mothers provided with the 25% casein diet that were also fostered to other mothers on a 25% casein diet were designated as members of the 25/25 (prenatally well-nourished) group and served as control subjects. This model was designed to investigate the effects of PPM during gestation with nutritional rehabilitation beginning at birth. On postnatal day (PND) 21, all rats were weaned and provided with a standard laboratory chow diet (Purina Mills Inc., Richmond, IN, United States; Formula 5001). Subjects were then pair-housed with same-sexed littermates and given *ad libitum* access to food and water. Research personnel were blind to dietary condition until the completion of data collection.

### *In vivo* Microdialysis

#### Stereotaxic Surgery

Male adult Long-Evans rats (90–120 days of age) were included in the present experiments. Only one animal from each litter was used here while littermates were assigned to other experiments [as reported, in part, by McGaughy et al. (2014)]. For surgery, rats were anesthetized with 2% isoflurane with oxygen (0.6 L/min). Lidocaine with epinephrine was injected subcutaneously at the site of the incision. Surgeries were done under aseptic conditions. During surgery, guide cannulae (CMA 12, CMA/Microdialysis AB, Acton, MA, United States) were implanted bilaterally into the vmPFC such that the tip of the guide cannula was located at the coordinates of A 3.2 mm; L ± 0.8 mm; DV 2 mm with reference to bregma according to the atlas of Paxinos and Watson (2005). After the holes for the guide cannulae were drilled, the guide cannulae were slowly lowered into place over a 3-min period. Guide cannula were affixed to the skull using three stainless steel screws with dental acrylic covering the screws and the guides.

#### Microdialysis Procedure

After 3 days of recovery following implantation of the guide cannulae, animals were placed in a large Plexiglas bowl with a collar attached by a guide wire to a suspension arm (CMA Microdialysis). All experiments took place between 0830 and 1200 h. On the day of the experiment, a 2 mm CMA-12 probe (CMA Microdialysis, Inc., N. Chelmsford, MA, United States) was placed into each cannula while the animal moved freely around the bowl. Artificial cerebrospinal fluid (artCSF; 147 mM NaCl, 1.26 mM CaCl_2_, 2.5 mM KCl, and 1.18 mM MgCl in sterile water) was perfused through the probe using a CMA/Microdialysis Syringe pump and a 1.0 ml gastight Hamilton syringe at a rate of 1.0 μl/min. After allowing 3 h for equilibration of the probes, samples (20 μl) were collected every 20 min for another 3 h. Animals were only used in one microdialysis experiment.

### Analysis of Monoamines

Samples were analyzed by high performance liquid chromatography with electrochemical detection (HPLC-ECD). The analytical system was an ESA Coulochem II (ESA Inc., Chelmsford, MA, United States) using a 3 μM 3 mm × 150 mm C-18 column (MD150, ESA Inc.). The mobile phase consisted of 90 mM sodium dihydrogen phosphate, 50 mM citric acid, 10% acetonitrile, 50 μM EDTA, pH of 3.0. This allowed for the measurement of 5-HT, DA, and NE with a sensitivity of 0.5 fmol/20 μl sample. The area under the curve (AUC) for samples were compared using computer software Chromperfect (Justice Laboratory Software, Palo Alto, CA, United States) with a regression analysis of AUC for three authentic standards (10^−8^, 5 × 10^−9^, 10^−9^ M) injected onto the column at the beginning of each experimental day. 5-HT, DA, and NE were verified by examining the voltammogram for standards against that determined using microdialysis samples.

### Histology

At the completion of each study, rats were perfused transcardially with 10% formalin under pentobarbital anesthesia and brains were removed. Perfused brains were then placed in 30% sucrose to provide cryoprotection. Brains were frozen in Tissue-Tek O.T.C. compound, sectioned in the coronal plane on a Leica CM1900 cryostat at 40 μm. Sections were then mounted and stained with cresyl violet for verification of the probe placement.

### Laterality

Previous work by Sullivan and coworkers (Sullivan and Dufresne, 2006; Sullivan et al., 2009a,b) and our lab (Staiti et al., 2011) have shown differences in levels of neurotransmitters in the left and right hemispheres dependent on age and sex of the rat. In order to determine the relationship between neurotransmitter levels in the two hemispheres we calculated a laterality index for each neurotransmitter. This index was calculated for each subject by determining the difference in the extracellular concentration of a specific neurotransmitter in left and right vmPFC and dividing by the sum of left and right concentrations, thus giving a value ranging between +1.0 and −1.0 (Staiti et al., 2011). Thus an index of zero indicates equal levels in each hemisphere, an index from 0 to +1 shows laterality to right and a level from 0 to −1 laterality on the left.

### Attentional Set Shifting Task (ASST)

In the present study, we report on correlations between neurotransmitter levels in the mPFC and behavior in an attentional task. The full report of the behavioral data has been published (McGaughy et al., 2014) but we summarize it as follows. Adult rats were trained to dig in pots for food reward. Animals were tested on a series of conditional discriminations with two stimuli per trial. The number of trials needed to reach criterion in the task was recorded. For each test, subjects were required to emit six correct consecutive responses. Trials to reach this level of criterion performance was the dependent measure at each stage. On the first discrimination, the stimuli varied on one dimension (simple discrimination). For all subsequent tests, stimuli differed in multiple dimensions; digging material, scent and texture of the pot. For the first five testing stages, subjects were reinforced for focusing on one attribute, e.g., odor, of a complex stimulus and to disregard other stimulus attributes, e.g., digging media. This same dimension was rewarded during reinforcement reversals and when a novel set of stimuli were introduced to facilitate formation of an attentional set. Once the set was formed, subjects were required to inhibit responding to this set to learn a previously irrelevant attribute of the complex stimuli now predicted reward (extra-dimensional shift). Previous work by McGaughy and co-workers has shown that NE in the PFC is important to the set-shifting portion of the task (McGaughy et al., 2008; Newman et al., 2008) so we determined how post-testing levels of NE, DA, or 5HT correlated with performance in the set-shifting portion of the task. Additionally, these neuromodulators have been shown to be critical to learning changes in reinforcement contingencies, so we also investigated the relationship of NE, DA, and 5HT to reversal learning (Clarke et al., 2005; den Ouden et al., 2013).

### Statistical Analysis

Extracellular concentrations of neurotransmitter in dialysate were converted to femtomoles per 20 μl sample. Analysis of variance (ANOVA) with day as a repeated measure was used to compare weights between the two nutritional groups (SigmaPlot^1^). Neurotransmitter levels across groups were analyzed using a three-way (ANOVA) with nutrition group, and cortical hemisphere as independent variables and time as a within subject variable. *Post-hoc* comparisons were performed between groups using Student-Newman-Keuls tests. The level of significance was set at *p* < 0.05 for all tests. Correlations between neurotransmitter levels and behavior in the ASST were done using linear correlational analysis (SPSS see text footnote^1^). Two-tailed tests were used for Pearson correlation analysis. Tables 1, 2 give F-values and significance between groups and analysis of correlation.

**Table 1:** *F*-values for two-way ANOVAs comparing neurotransmitter levels in 6/25 and 25/25 groups.

	Diet	Hemisphere	Diet × Hemisphere
NE	1.874	0.009	4.998^∗^
DA	0.032	7.433^∗^	1.906
5-HT	4.928^∗^	3.459	7.249^∗^

^*^p < 0.05.

**Table 2:** Correlations (*r*^2^-values) between performance on the ASST and basal 5-HT, DA, and NE levels determined by microdialysis in a sub-set of animals that were first assessed in the ASST (total reversals and extradimensional shift) and then assessed in the microdialysis experiment.

	25/25 (*N* = 6)		6/25 (*N* = 6)	
	Total reversals	Extra-dimensional shift	Total reversals	Extra-dimensional shift
ID/ED trials to criterion	46.7 ± 2.7	15.8 ± 2.3	54.7 ± 4.36	25 ± 3.9
5-HT Left hemisphere	0.649	0.821^∗^	−0.482	0.514
5-HT Right hemisphere	0.666	−0.366	−0.403	0.093
Dopamine L	−0.087	0.511	0.033	0.804
Dopamine R	0.250	−0.798	−0.439	0.794
Norepinephrine L	0.031	0.443	0.309	−0.071
Norepinephrine R	−0.846^∗^	−0.392	−0.252	−0.129

*Values for ID/ED trials to criterion are mean ± SEM [as reported in McGaughy et al. (2014)]. Values for correlations are r-squared values; ^∗^p < 0.05.*

## Results

### Changes in Basal Levels of Monoamines

Following post-mortem histology, data from two animals in the 6/25 group and three animals in the 25/25 group were eliminated as probe placement was outside the vmPFC. All animals that were included in the present study showed microdialysis probe placement within the vmPFC.

Rats exposed prenatally to a 6% protein diet (6/25 animals) did not differ significantly from the 25% protein control rats (25/25 animals) in initial litter size or postnatal mortality. However, they weighed significantly less than controls between PND 40 and PND 90, after which the weight difference was no longer present [Supplementary Figure S1; McGaughy et al. (2014)].

**FIGURE 1:**
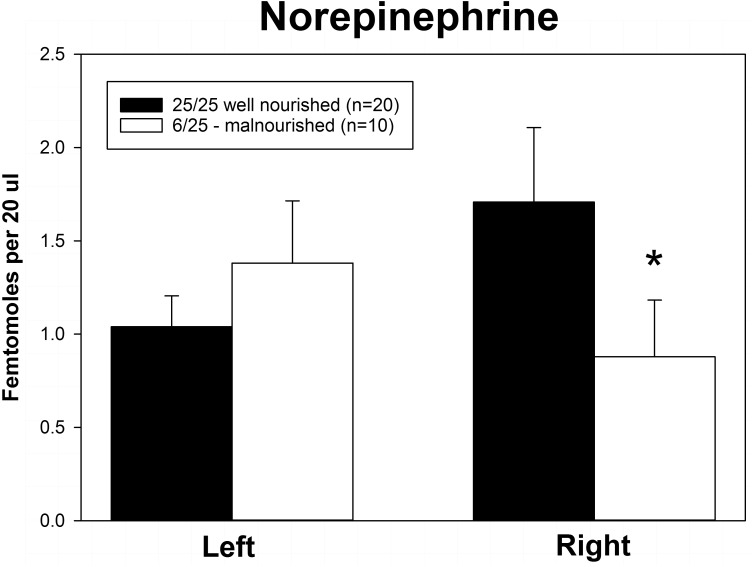
Basal levels of norepinephrine in left and right ventral medial prefrontal cortex of adult male rats exposed to prenatal protein malnutrition (6/25 malnourished) compared to well-nourished controls (25/25 well nourished). NE levels in the right vmPFC of malnourished rats were significantly lower than in well-nourished animals (^∗^*p* < 0.05) but did not differ significantly on the left.

**FIGURE 2:**
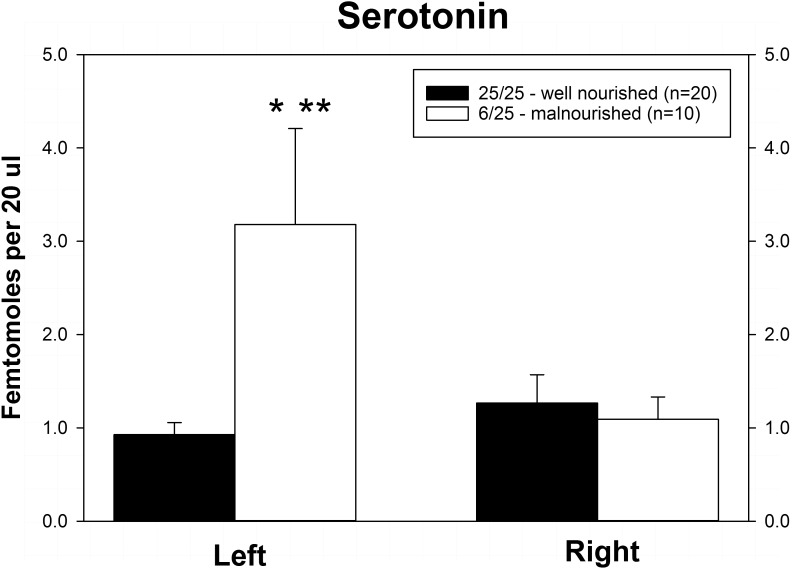
Basal levels of serotonin (5-HT) in left and right ventral medial prefrontal cortex of adult male rats exposed to prenatal protein malnutrition (6/25 malnourished) compared to well-nourished controls (25/25 well nourished). 5-HT levels in the left hemisphere were significantly greater in 6/25 animals than in well-nourished 25/25 controls (^∗^*p* < 0.05) and also greater than 6/25 values in the right vmPFC (^∗∗^*p* < 0.01).

**FIGURE 3:**
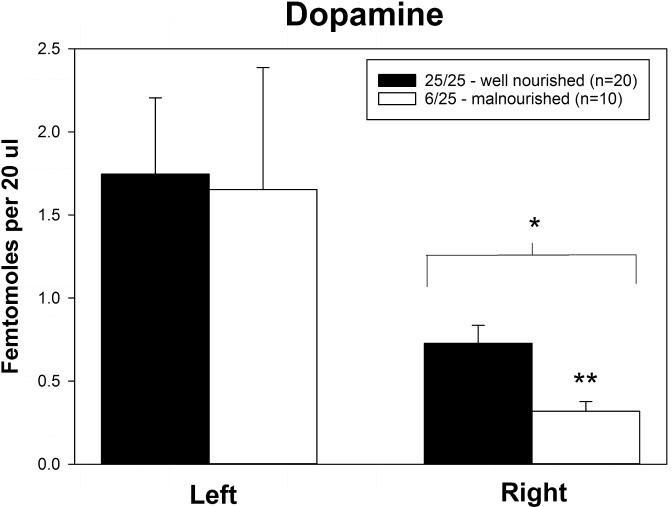
Basal levels of dopamine in left and right ventral medial prefrontal cortex of adult male rats exposed to prenatal protein malnutrition (6/25 malnourished) compared to well-nourished controls (25/25 well nourished). DA levels in the right vmPFC of malnourished rats were significantly lower than in well-nourished animals (*p* < 0.05). Furthermore, DA levels in the right vmPFC of malnourished rats were lower than DA levels in the left vmPFC. ^∗^Significantly different from left hemisphere, ^∗∗^Significantly different from well nourished group.

Rats exposed to PPM differed in the basal concentrations of NE, 5-HT and DA, and these differences were lateralized. Basal levels of NE were significantly decreased in the right vmPFC of 6/25 animals but not in the left vmPFC which was reflected by a significant interaction of nutrition group x hemisphere (Figure 1 and Table 1). Basal extracellular serotonin levels were increased in the left vmPFC in 6/25 rats, but unchanged in the right hemisphere relative to 25/25 animals (Figure 2 and Table 1). In regard to serotonin levels, there was a significant nutrition group effect, and a significant interaction between nutrition group and hemisphere (Table 1), but no difference in levels between the two hemispheres. DA levels in the vmPFC were significantly decreased in the right hemisphere in 6/25 rats compared to 25/25 rats (Figure 3 and Table 1). DA levels differed between the hemispheres but there was not an effect of diet or interaction between diet and hemisphere. Both NE and DA levels in the left hemisphere were similar regardless of nutrition group. Since each microdialysis sample contained the extracellular concentration of the three neurotransmitters during one 20-min epoch, we also looked at the correlations between the concentrations of DA, 5-HT, and NE in the vmPFC during each 20-min period in each animal in both the left and right hemisphere of the vmPFC. We did not find any significant correlations between the changes in the levels of these neurotransmitters over the time course of the experiment. Thus, for instance, the changes in 5-HT levels that we observed during the experiment did not correlate with changes in either NE or DA.

**FIGURE 4:**
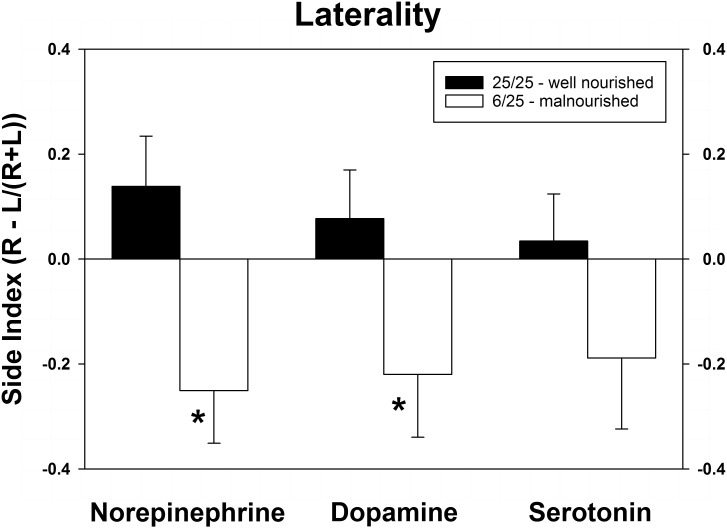
Changes in cerebral laterality in extracellular concentrations of NE, DA, and 5-HT following exposure to prenatal protein malnutrition comparing 6/25 malnourished rats with 25/25 well-nourished adult rats. NE and DA laterality shifted from the right hemisphere to the left hemisphere in 6/25 animals,^∗^*p* < 0.05.

As shown in Figure 4, PPM significantly altered the laterality index for NE and DA. Although the index for serotonin was altered in a similar direction (toward the left hemisphere) as the other two neurotransmitters, this difference did not achieve significance. Interestingly, the balance of all three neurotransmitters was shifted from the right to the left vmPFC.

### Correlations of Basal Efflux and Prior Cognitive Testing

**FIGURE 5:**
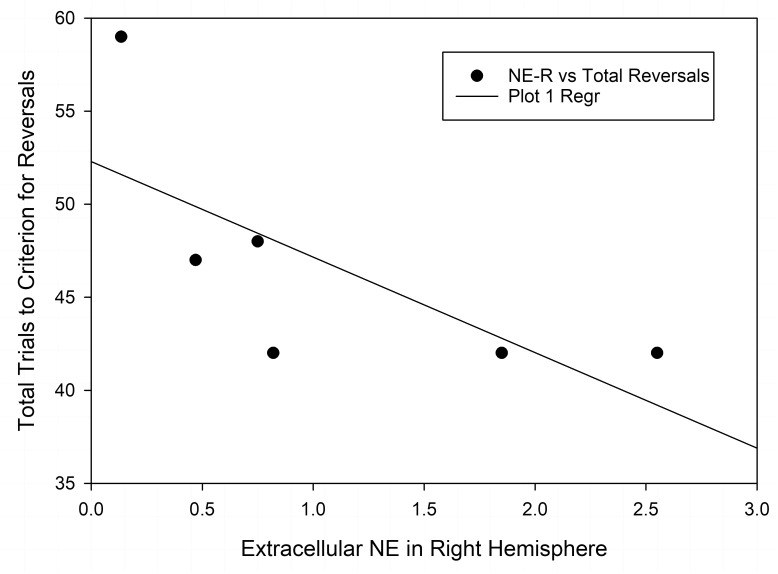
Correlation of extracellular NE in the right vmPFC and total reversals in the ASST. The r-squared for the Pearson correlation is –0.846, *p* < 0.05.

Finally, we also compared the changes in neurotransmitter levels with the results of the test of set-shifting using a subset of animals (*n* = 6 in each nutrition group) that were tested in an ASST [as reported by McGaughy et al. (2014)] before being assigned to the microdialysis experiment. Table 2 shows results of correlational analyses comparing the trials to criteria for reversals and the extradimensional shift in the ASST with basal concentrations of NE, DA, and 5-HT in each hemisphere. The number of trials to criteria for reversals had a significant negative correlation with extracellular NE in the right hemisphere of well-nourished animals (r-squared = −0.846, *p* < 0.05, Figure 5 and Table 2). There was also a significant correlation between 5-HT in the left vmPFC and trials to criteria for the extra-dimensional shift of well-nourished animals (r-squared = 0.821, *p* < 0.05). However, there was no significant correlations between performance on the ASST and neurotransmitter levels in the prenatally malnourished 6/25 animals. Due to the low numbers in the sample this association is only tentative and needs to be replicated but is of interest given the role of NE and the mPFC in the ASST task (McGaughy et al., 2014; Mokler et al., 2017).

## Discussion

### Changes in Dopamine

Here we found a decrease in DA in the right vmPFC of 6/25 animals compared to well-nourished controls (25/25), while there was no difference in left vmPFC. This confirms a similar decrease in extracellular DA in the right vmPFC reported in an earlier study in this laboratory (Mokler et al., 2007). Additionally, Kehoe et al. (2001) reported a decreased DA in hippocampus of malnourished animals in a similar model of PPM, suggesting that there may be widespread decrease of dopaminergic function after PPM.

The role of prefrontal DA in mediating a response to acute stress has been shown to be lateralized. Non-specific lesions to the right infralimbic cortex (IL) suppress the stress response. In contrast, when the lesions are selective for DA in the right IL PFC there is an enhanced stress response (Sullivan and Gratton, 2002; Sullivan, 2004). A number of studies by our group have documented an altered stress response in animals exposed to PPM (Trzcinska et al., 1999; Mokler et al., 2003, 2007) which may therefore be a result of the perturbations in cortical DA. Rats exposed to PPM (Mokler et al., 2007) showed a lack of effect of a restraint stress on DA in the right vmPFC. In contrast, well-nourished controls show a 50% increase in DA efflux after acute stress. Kehoe et al. (2001) have also reported a decreased corticosterone response to stress in animals exposed to prenatal protein dietary restriction. Similar findings have been reported in human populations. For example, in their study of stunted children (growth retardation) in Nepal, Fernald et al. (2003) reported a blunted cortisol response in these children. Overall, these results point to DA in the vmPFC as a potentially important mediator of behavioral responses in the context of stress.

### Changes in Norepinephrine

In the present study, we examined changes in extracellular NE in the vmPFC of prenatally malnourished animals due to the important role of NE in the PFC in attention (Mokler et al., 2017) (Newman et al., 2019). We report a decrease in extracellular NE in the right but not the left vmPFC in PPM animals, a finding that parallels the effects of PPM on DA. In their study of animals who were chronically exposed to an 8% protein diet both prenatally and after birth, Stern et al. (1975) reported an increase in NE in gross brain dissection of telencephalon and diencephalon at some ages but not at day 60, the closest time point to the present study. However, using a model of PPM similar to ours, Soto-Moyano et al. (1999, 2005) reported increased neocortical NE and α2a – adrenoreceptors in rat pups exposed to an 8% protein diet prior to and during pregnancy. These investigators also reported a decrease in NE release as well as increased α2a – adrenoreceptors in the neocortex of adult PPM rats. The decreased extracellular NE in the right PFC reported in the current study may be important for understanding the cognitive rigidity that we have seen in prenatally malnourished animals (McGaughy et al., 2014; Peter et al., 2016; Newman et al., 2019). McGaughy and co-workers (Newman et al., 2019), however, report that in this same model of prenatal malnutrition, there was a bilateral decrease in noradrenergic axons in vmPFC. It is unclear how this finding will be reconciled with our current finding of a decrease in NE in only the right vmPFC. Future studies are needed to determine if other characteristics of noradrenergic axons are changed, e.g., density of varicosities and if this effect is lateralized. It should be pointed out that in earlier studies by our group, we have also seen an increase in extracellular hippocampal 5-HT (Mokler et al., 1999, 2003) despite a decrease in serotonergic fibers innervating the hippocampal formation suggesting more complex effects on metabolism and/or release (Blatt et al., 1994). More work needs to be done to clarify the mechanisms involved in an increase in extracellular neurotransmitter in the face of a decreased in axon density.

To assess the behavioral significance of our findings on NE, we examined the association between extracellular neurotransmitter levels and performance on the ASST, an attentional set shifting task sensitive to PFC manipulations. There was a significant correlation between performance on total reversal trials and cortical NE in the right hemisphere of the vmPFC in well-nourished but not malnourished subjects. Higher levels of NE in the right vmPFC of well-nourished animals were correlated with lower trials to criteria in the ASST task. In malnourished animals the correlation was low (r-squared = −0.252), which together with the significant reduction in basal levels of NE in the right vmPFC further indicates the importance of NE in the ASST behavioral task. Thus, even in the absence of developmental changes induced by PPM, well-nourished animals also show a relationship between cortical NE and reversal learning. In the full cohort of animals tested in the ASST task, malnourished animals had significantly higher total reversal trials than well-nourished animals (McGaughy et al., 2014) Because reversal learning relies on the orbitofrontal cortex and the mPFC, this may reflect impaired cortico-cortical connections in malnourished animals. We are planning to investigate the connectedness of prefrontal circuits after malnutrition and to directly interrogate the orbitofrontal cortex. It also should be noted that there was a low correlation between extracellular NE in the left vmPFC and total reversals, suggesting that the right vmPFC may be more involved in reversal learning. Further research in this area is also underway. It should also be pointed out that microdialysis was not performed in animals during the performance of the task, a critical next step in assessing the dynamic role of NE during attentional performance. These data and the work of Newman et al., 2019 suggest an important role of the PFC and NE in the attentional problems of malnourished animals.

### Changes in Serotonin

One of the most consistent neurochemical changes which we have observed in adult male rats exposed to PPM is the alteration of the serotonergic system of the forebrain. Neurochemical analysis of whole brain (Chen et al., 1992, 1995), hippocampus (Mokler et al., 1999, 2003), and PFC (Mokler et al., 2007) of animals exposed to PPM have demonstrated elevations in 5-HT levels. In the present study we also found an increase in the extracellular levels of 5-HT in the left vmPFC in malnourished animals, while the levels of 5-HT in the right vmPFC were unaffected by PPM. Serotonin has been linked to impulsivity, specifically in cortico-striatal circuits involving the PFC (Dalley and Roiser, 2012). 5-HT2A receptor antagonists such as M100907 reduce impulsivity in rats selected for this trait (Anastasio et al., 2015). Thus, the increase in 5-HT observed in animals exposed to PPM may explain our observation of increased impulsivity in our model. Almeida et al. (1996b) reported in this model of malnutrition that malnourished animals showed heightened impulsivity or reduced anxiety on the elevated plus maze. This, in addition to the cognitive rigidity that we have reported in this model (McGaughy et al., 2014; Newman et al., 2019), adds to the phenotype of the prenatally malnourished animals.

There was also a significant positive correlation between 5-HT levels in the left vmPFC and trials to criterion for the extradimensional shift in well-nourished animals. This correlation was not significant in malnourished animals. Thus, in well-nourished animals increased 5-HT in the vmPFC is associated with increased trials to criterion in the extra-dimensional shift. It has been reported that antagonists at the 5-HT_6_ receptor improve performance in the ASST (Hatcher et al., 2005). However, Lapiz-Bluhm et al. (2010) showed that global 5-HT depletion did not affect ED in the ASST. While much of the work on 5-HT role in reversal learning and performance in the ASST is focused on the orbital frontal cortex, more research is needed to determine the role of 5-HT in the vmPFC in this task.

### Hemisphere Asymmetries in Monoamines and Interaction With PPM

Of particular interest are the hemispheric differences in extracellular concentrations of the neurotransmitters. In each case the differences between 6/25 (prenatally malnourished) and 25/25 (well nourished) animals were limited to one side of the cortex. In the case of DA and NE the changes seen in 6/25 animals were limited to the right hemisphere whereas with 5-HT the changes were seen in the left hemisphere. In our previous observations we also found a decrease in basal extracellular DA in the right mPFC (Mokler et al., 2007). Although our previous study used Sprague-Dawley rats rather than the Long-Evans rats used in the present study, the degree of DA decrease was similar demonstrating the robustness of the nutrition effect across different rat strains. Furthermore, in agreement with our current findings of a lack of 5-HT changes in 6/25 animals in the right mPFC, in that earlier study which looked only at the right hemisphere, we did not see changes in 5-HT. In this same model of prenatal malnutrition, Lister et al. (2006) have also looked at hemispheric differences in the hippocampal formation. They reported that there is a smaller number of neurons in the right CA1 and CA2/CA3 subfields of the hippocampus; PPM decreases those numbers in both hemispheres.

Interestingly, deficits in right medial PFC function associated with attention have also been reported in a normative animal model. In the five choice serial reaction time test (5-CSRTT), a test of attention and impulsivity, adult rats with attention deficits had higher 5-HT in left PFC and lower DA in right PFC (Davids et al., 2003), similar to our current findings. The findings of an increase in 5-HT in the left vmPFC are in line with our findings in the current study and may be related to the findings of increased impulsivity (or decreased anxiety) in the elevated plus maze and elevated *T*-maze in animals exposed to PPM (Almeida et al., 1996b,c). Overall, more research is needed focused on the significance of hemispheric differences in the rat PFC, but the present results point to the importance of assessing such differences in PPM animals.

Finally, while little is known about lateralization of neuromodulatory systems in human brain, there have been indications of differences in the two hemispheres with regard to attentional problems. Patients with attentional deficit disorder have low extracellular DA in the right basal ganglia compared to matched controls (Davids et al., 2003). Hence in addition to the importance of comparing both hemispheres future studies should be aimed at determining how the neurochemical lateralization reported here in rats pertains to behavior, as well as, hemispheric asymmetries in human brain function.

## Summary and Conclusion

In summary, we report significant changes in the balance of the monoaminergic neurotransmitters (serotonin, DA and NE) in the vmPFC of adult rats exposed to PPM. The changes in right vmPFC DA and NE may, in part, play a role in the cognitive inflexibility reported in this model of PPM (McGaughy et al., 2014) (Newman et al., 2019). Furthermore, the increased extracellular 5-HT in the left vmPFC may also be associated with the increased impulsivity (or less anxiety) in this animal model. The association of performance in the attentional set shifting task and levels of NE in the right vmPFC and 5-HT in the left vmPFC of well-nourished animals suggest the need for further studies on the hemispheric roles of these neurotransmitters in learning. While the functional significance and underlying alterations in brain circuits suggested by the hemispheric difference reported here is puzzling, these observations make it imperative that future studies compare neurobiological markers in both the right and left hemispheres using methods like dual probe microdialysis during behavioral assessment so that firm correlations can be evaluated. Further work is needed to assess the anatomical changes which lead to the differences in extracellular neurotransmitter seen in this study.

The present data, as well as previous studies of the effects of PPM in altering the developing and adult brain of the rat (Stern et al., 1975; Chen et al., 1992, 1995; Blatt et al., 1994; Mokler et al., 1999, 2003, 2007; Soto-Moyano et al., 1999, 2005; McGaughy et al., 2014; Newman et al., 2019), suggest a reprogramming of the brain. This reprogramming may be adaptive in allowing the animal to focus more on the need to find nutrition in a nutritionally sparse environment, which is reflected in the reported cognitive inflexibility. More work is needed to determine if, in fact, these changes in brain function may benefit the individual when food scarcity occurs. However, these behavioral changes may not be adaptive after nutrition privation is reversed.

## Data Availability

The datasets generated for this study are available on request to the corresponding author.

## Author Contributions

All authors contributed to the design of the experiments and breeding, and were involved in manuscript writing and review. DM, DB, and PM were responsible for the surgeries and microdialysis experiments. DM, DB, and JM were responsible for the behavioral experiments. DM, JM, PM, and JRG were responsible for data analysis.

## Conflict of Interest Statement

The authors declare that the research was conducted in the absence of any commercial or financial relationships that could be construed as a potential conflict of interest.

## Footnotes

1 systatsoftware.com

## Supplementary Material

FIGURE S1 The average weight of all pups after weaning, by diet group, excluding litters with less than four pups and animals on food restriction for behavioral testing. ^∗^*p* < 0.05 different compared to 6/25 (malnourished) animals, ANOVA.
