# Supplementary figures and images for: Prenatal Protein Malnutrition Leads to Hemispheric Differences in the Extracellular Concentrations of Norepinephrine, Dopamine and Serotonin in the Medial Prefrontal Cortex of Adult Rats
Source: Front Neurosci. 2019 Mar 5;13:136. doi: 10.3389/fnins.2019.00136 (PMC6411819; doi:10.3389/fnins.2019.00136)

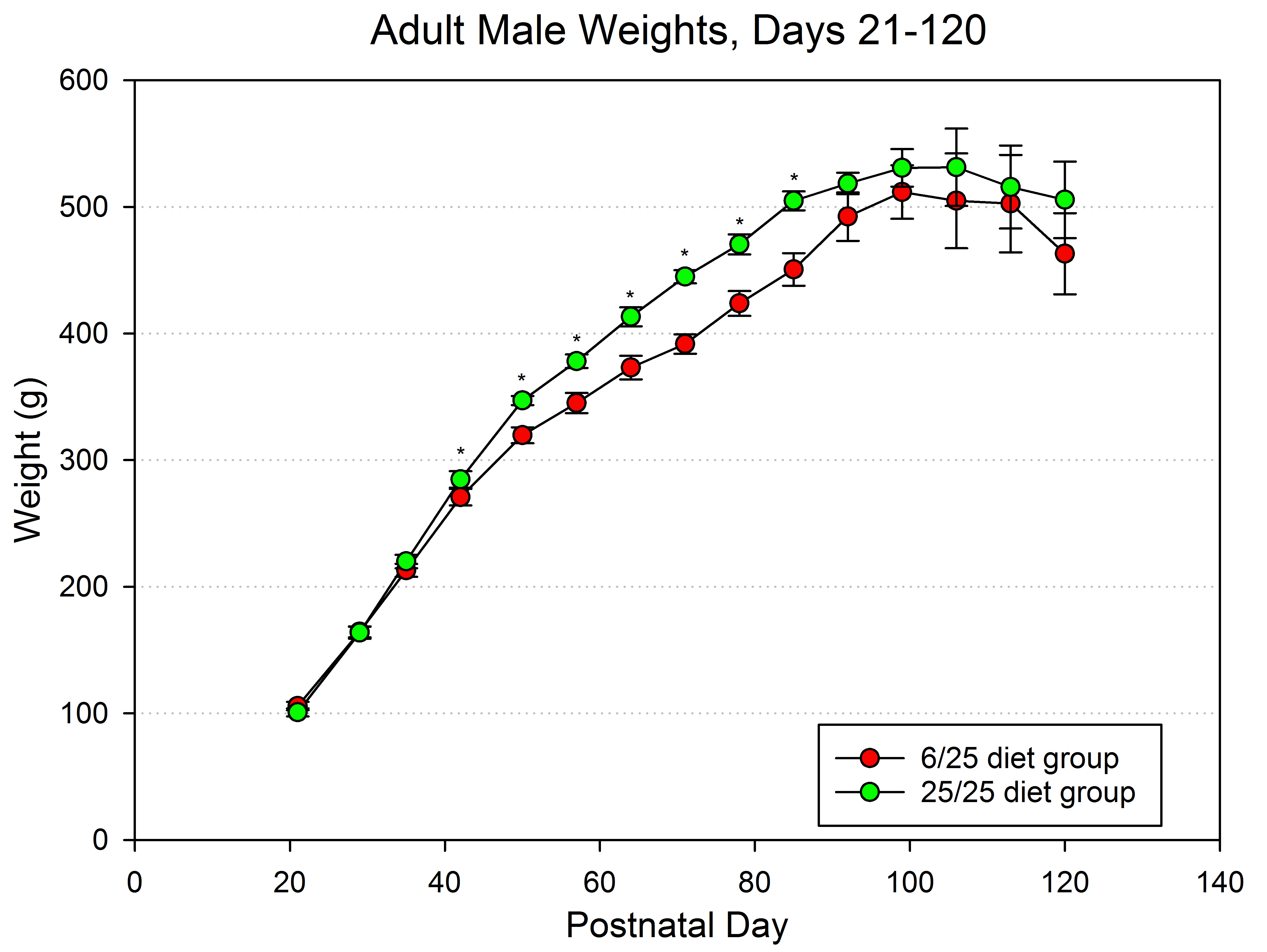

Supplement: FIGURE S1 — The average weight of all pups after weaning, by diet group, excluding litters with less than four pups and animals on food restriction for behavioral testing. ∗p < 0.05 different compared to 6/25 (malnourished) animals, ANOVA. [file Image_1.JPEG]
